# Hereditary Pancreatic and Hepatobiliary Cancers

**DOI:** 10.1155/2011/154673

**Published:** 2011-06-28

**Authors:** Ashraf Haddad, Gopal C. Kowdley, Timothy M. Pawlik, Steven C. Cunningham

**Affiliations:** ^1^Department of Surgery, Saint Agnes Hospital, Baltimore, MD 21229, USA; ^2^Department of Surgery, The Johns Hopkins Hospital, Baltimore, MD 21231, USA

## Abstract

Hereditary etiologies of pancreatic and hepatobiliary cancers are increasingly recognized. An estimated >10% of pancreatic and increasing number of hepatobiliary cancers are hereditary. The cumulative risk of hereditary pancreatic cancer ranges from measurable but negligible in cystic fibrosis to a sobering 70% in cases of hereditary pancreatitis. Candidates for pancreatic cancer surveillance are those with a risk pancreatic cancer estimated to be >10-fold that of the normal population. Screening for pancreatic cancer in high-risk individuals is typically performed by endoscopic ultrasound and should begin at least 10 years prior to the age of the youngest affected relative. Disease states known to be associated with increased risk of hepatocellular cancer include hereditary hemochromatosis, autoimmune hepatitis, porphyria, and *α*1-antitrypsin deficiency, with relative risks as high as 36-fold. Although much less is known about hereditary bile-duct cancers, Muir-Torre syndrome and bile salt export pump deficiency are diseases whose association with hereditary carcinogenesis is under investigation.

## 1. Introduction

Hereditary etiologies of carcinogenesis have been increasingly recognized over the past century. In 1889, Billroth was one of the first to recognize the occurrence of multiple primary malignant neoplasms [1] and Lynch et al. in 1967 one of the first to draw attention to the etiologic role of hereditary factors in their occurrence [2]. Since then, at least 78 identified genetic syndromes of hereditary cancer have been described [3] and many more syndromes undoubtedly remain to be identified.

Recent molecular and genetic advancements have brought international attention to pancreatic cancer (PC), with an estimated >10% of cases being hereditary in etiology. Although less common then hereditary PC, hereditary hepatobiliary cancers are also increasingly recognized. 

## 2. Pancreas Cancer

PC is a lethal disease, with 277,000 new cases diagnosed per year globally [4]; in the USA, it was estimated to be 43,140 new cases of pancreatic cancer and 36,800 deaths from the disease in 2010 [5]. The definition of familial, or hereditary PC (HPC), the vast majority of which is pancreatic ductal adenocarcinoma (PDAC), is generally accepted to be defined as PC occurring in families with ≥2 first-degree relatives (FDRs) [6]. 

Six years after the landmark 1967 publication by Lynch et al. on the role of hereditary factors in the occurrence of multiple primary malignant neoplasms [2], a kindred of four siblings with pancreas adenocarcinoma was described [7]. Over the next two decades, several more publications highlighted the importance of the hereditary form of pancreatic adenocarcinoma, not only within a single generation but also across multiple generations and families [8, 9] (Table 1).

Population-based case-control studies have quantified the increase in risk for individuals from families with convincing family histories of PDAC. For example, a study in 1990 from the Hereditary Cancer Institute at Creighton University identified familial clustering among 47 cases of pancreatic cancer in 18 nuclear families with two or more first-degree relatives affected [10]. In this study, while only 0.7% of controls had positive family histories of the disease, 6.7% of PDAC cases had positive family histories. Ghadirian et al. [11] conducted a similar study on 179 French-speaking patients in Montréal and found that 7.8% of the patients who had PDAC had a family history of the disease compared with only 0.6% of the control population representing a 13-fold increase. Dergham et al. [13] reported a similar figure of 9% of pancreatic cancer patients having a family history. In a Northern Italy study including >300 patients with PDAC, a family history of pancreatic cancer was associated with an increased relative risk of 3 for the development of pancreatic cancer (RR = 3) [12].

Larger, national studies [14, 15] and a meta-analysis of studies [17] of thousands to tens of thousands of patients with PC similarly have revealed an increased relative risk ranging from 1.5 to 3.4 based on family history of PC. The risk of PC in family members of those who have PC is likely best estimated by analysis of the National Familial Pancreas Tumor Registry at Johns Hopkins Hospital [16]: among 5179 individuals in 838 kindreds, the risk (standardized incidence ratios) of persons with 1, 2, or 3 FDRs with PC was 4.6, 6.4, and 32, respectively (Table 1). 

In addition to familial PC, among other genetic defect(s) which remain to be discovered, there are at least five well-described inherited profiles with known genetic defects that substantially increase the carrier's cumulative lifetime risk for developing HPC (Table 2).

### 2.1. Specific Diseases with Inherited Predisposition for Pancreatic Cancer

#### 2.1.1. Hereditary Pancreatitis

Hereditary pancreatitis (HP) is an autosomal dominant disease that presents with episodes of acute pancreatitis in early childhood, as early as 5 years of age. As a result of such early onset of acute pancreatitis, most of these patients develop chronic pancreatitis in their second decade of life, unlike chronic alcoholic pancreatitis which presents later in life. Chronic HP is further distinguished from chronic alcoholic pancreatitis by the equal gender ratio, but is otherwise largely similar to nonhereditary pancreatitis (Table 3) [34].

Any difficulty differentiating HP from the nonhereditary form on the basis of biochemical and laboratory differences was mitigated by the discovery of a genetic difference: a 1996 study of five kindreds with hereditary pancreatitis revealed that an arginine-histamine substitution at residue 117 of the cationic trypsinogen gene, *PRSS1*, was associated with the phenotypic expression of acute pancreatitis due to failure of the affected trypsinogen, leading to autodigestion of the pancreas [35]. A second mutation was later described—a single A to T mutation—to be associated with a less severe form of hereditary pancreatitis [36]. 

Hereditary pancreatitis is associated with a markedly increased risk (53-fold) for developing PC (Table 2), which usually develops after several decades of pancreatitis especially in those with paternal mode of inheritance [18]. Smoking, a known risk factor for PC, was found to have an even more pronounced detrimental effect on those with hereditary pancreatitis: in a cohort of 497 patients, smoking was found to double the risk for PC and to be associated with a 2-decade-earlier onset of PC compared with nonsmoking status [37].

#### 2.1.2. Familial Atypical Multiple Mole Melanoma Syndrome

The familial atypical multiple mole melanoma syndrome (FAMMM) is an autosomal dominant syndrome associated with an increased risk of cutaneous melanoma, dysplastic nevi, and PC, among other neoplasms. The association of the FAMMM syndrome with PC was described in 1990 by Bergman et al. [21], who studied nine families (200 individuals) with FAMMM in the Netherlands, reporting nine cases of PC, which has a 13.4-fold increased risk compared with the general population.

The *CDKN2A* gene, also known as *P16*, is a tumor-suppressor gene with various mutations implicated in the development of FAMMM as well as other systemic cancers. Studies of FAMMM individuals whose *P16/CDKN2A* was rendered dysfunctional by mutations have noted that the risk of PC was increased 15- to 38-fold [22, 38].

#### 2.1.3. Hereditary Breast and Ovarian Cancer Syndrome and PALB2 Loss

Mutations of *BRCA1 *and* BRCA2*, common in hereditary breast and ovarian cancer (HBOC) syndrome, predispose carriers to developing breast, ovarian, and a variety of other cancers including PC. Sequencing of *BRCA2* in individuals with HPC from North American [39] and European [40] HPC registries have revealed deleterious mutations in nearly one-fifth of these individuals, making *BRCA2* the most common genetic defect in HPC. Loss of *BRCA2* in HPC has been observed to confer an increased risk of PC as high as nearly 9-fold [24]. The role of *BRCA1* seems to be less pronounced, but still measureable at a 2- to 3-fold increased risk of PC in carriers of *BRCA1* mutations [25, 26]. Goggins et al. at Johns Hopkins University [41] studied 41 unselected adenocarcinomas of the pancreas, 15 of which had allelic loss at the *BRCA2* locus and 4 of those (9.8% overall) had a second-allele abnormality. Three of these four cancers (7.3% overall) were considered germ-line mutations (with confirmation in normal tissue) suggesting that the rate of germ-line *BRCA2* mutations in apparently sporadic pancreatic cancer may be as high as in breast or ovarian cancer [41].

The recently recognized partner and localizer of *BRCA2*, *PALB2*, is also frequently lost in HPC, at a rate of approximately 3% in recent North American [42] and European [43] sequencing studies of HPC kindreds, making it the second most commonly lost gene in HPC. Knowledge of such mutations as *BRCA2 *and* PALB2* has major therapeutic implications; PC in this group of patients has been shown to be exquisitely sensitive to DNA cross-linking agents such as mitomycin C (MMC) [44].

#### 2.1.4. Peutz-Jeghers Syndrome

Peutz-Jeghers syndrome (PJS) is an autosomal dominant syndrome associated with loss of *STK11/LKB1* gene function and is characterized by hamartomatous polyps in the gastrointestinal tract and pigmented skin lesions on the lips, oral mucosa, and digits. In a large retrospective cohort study of 34 PJS patients identified over 50 years of Mayo Clinic records, the overall risk for developing cancer in affected individuals was 9.9. The relative risk of developing gastrointestinal cancers was 50.5, and the risk was 5-fold higher in women (RR 151) than men (RR 30) [45]. The risk for developing PC was reported to be 5% at the age of 40 and 8% at the age of 60 in a study of 240 international PJS patients possessing the *STK11* mutation [27]. Of note, all pancreatic cancers in that study were diagnosed between the age of 34 and 49 years [27]. That multi-institutional effort was recently extended to include 419 PJS patients, 297 with an identified *STK11/LKB1* germ-line mutation [46], with similar results in the risk of PC (3- and 7-fold risk at 40 and 60 years of age, respectively.

#### 2.1.5. Lynch Syndrome

Lynch syndrome, also known as hereditary nonpolyposis colorectal cancer (HNPCC), is an autosomal dominant condition associated with mutations in DNA mismatch repair (MMR) genes including *MLH1*, *MSH2*, *MSH6*, *PMS2*, and others. The resulting compromise of DNA maintenance and repair leads to the accumulation of errors in the genome manifested in microsatellite instability and loss of normal tumor-suppressor function. Whereas Lynch I syndrome is comprised only of colorectal cancers, Lynch II has been characterized to include a number of extracolonic cancers including PC. 

Kastrinos et al. [30] analyzed the data on 6,342 individuals from 147 families with MMR gene mutations from two major US cancer centers and found that 21.1% of the families reported a case of pancreatic cancer. The cumulative risk of pancreatic cancer in these individuals was 1.3% at age 50 and 3.7% at age 70, which corresponds to a an 8.6-fold increase compared to the general population [30].

### 2.2. Surveillance and Screening for PC

PC, specifically PDAC, is generally a lethal disease, and even at high-volume institutions, the median survival following resection of PDAC is less than 20 months and the 5-year survival is only 20% [47]. However, when very small, very favorable cases of PC are selected, long-term survival is possible, with 4- and 5-year survivals of 78% and 59% reported [48, 49]. Usually, such cases are incidentally and fortuitously discovered at an early stage. While screening the low-risk general population for PC would be associated with an unfavorable risk/benefit ratio (due the low overall incidence of PC and to the lack of a screening test that is readily available, noninvasive, and accurate), screening a population at very high (>10-fold [50]) risk may offer an opportunity to cure an otherwise uncurable cancer if discovered early.

Investigators at the University of Washington were among the first to describe the use of prospective screening and surveillance for high-risk individuals [51]: 7 of 14 individuals from 3 high-risk families who were screened with endoscopic ultrasonography (EUS), endoscopic retrograde cholangiopancreatography (ERCP), computed tomography (CT), and serum CEA and CA19-9 were found to have high-risk lesions based on concerning features on EUS and ERCP features. Pancreatectomy was therefore recommended and performed in all 7 patients, and all 7 patients had widespread dysplasia (PanINs), but no cancer or normal pancreas parenchyma was found in any of the specimens [51]. In a follow-up study at the same institution, Kimmey et al. screened 46 high-risk patients with EUS [52]: 13 patients had abnormal findings, 12 of whom underwent pancreatectomy with all 12 specimens showing widespread dysplasia (PanIN). 

Canto et al. [53] at Johns Hopkins similarly reported on 38 high-risk (most with ≥3 relatives with PC) individuals who underwent screening with EUS and, if abnormal, then biopsy, ERCP, and CT. Resection was offered to and performed on 6 patients with a mass seen on EUS. On final pathology, 4 patients had a benign lesion, one patient had an IPMN, and one patient had PDAC [53]. Other centers around the world, including Germany, the Netherlands, and the US [54–56] have similarly begun screening programs.

Recommendations regarding screening and surveillance are in evolution. The University of Washington currently recommends surveillance to the following: (1) individuals with 2 or more first-degree relatives with PC, (2) individuals with one first-degree relative with PC diagnosed under the age of 50, (3) individuals with 2 or more relatives with pancreatic cancer, one of whom had PC at an early age, and (4) individuals with a genetic disorders, such as PJS and FAMMM [57]. Screening recommendations of the Fourth International Symposium of Inherited Diseases of the Pancreas [50], including both University of Washington and Johns Hopkins investigators, are slightly more stringent (Table 4) and include anyone deemed to have a risk of PC ≥10-fold the general population. As such, candidates for screening and surveillance include those with FAMMM, PJS, HP, or ≥3 first-degree relatives with PC, individuals with ≥3 first-, second-, or third-degree relatives with PC (at least one of whom is a first-degree relative), any member of a PJS family, those carrying mutations of *BRCA1*, *BRCA2*, or an MMR gene, and with at least one first- or second-degree relative with PC, and candidates with 2 relatives with PC in the same lineage (directly connected), at least one of whom is a first-degree relative of the candidate [50].

After deciding which patients to screen, the questions of how and when to screen remain. In addition to EUS and ERCP, magnetic resonance imaging with cholangiopancreatography (MRI/MRCP) has more recently gained increasing interest as a screening modality. Vasen et al. [58] used MRI/MRCP to screen high-risk individuals with *P16-leiden* mutations. After a 4-year median follow-up period, out of 79 individuals screened, pancreatic cancer was diagnosed in 9% and precursor lesions in 11% [58]. Whichever screening tool is employed, a screening program should take place only in the setting of a high-volume center and with full informed consent. Patients who are not willing to undergo pancreatectomy for suspicious lesions identified on screening should not undergo screening. Care must also be taken to exclude patients with a recent history of pancreatitis or heavy alcohol intake, since EUS findings are similar in that population [59]. 

**Figure 1:**
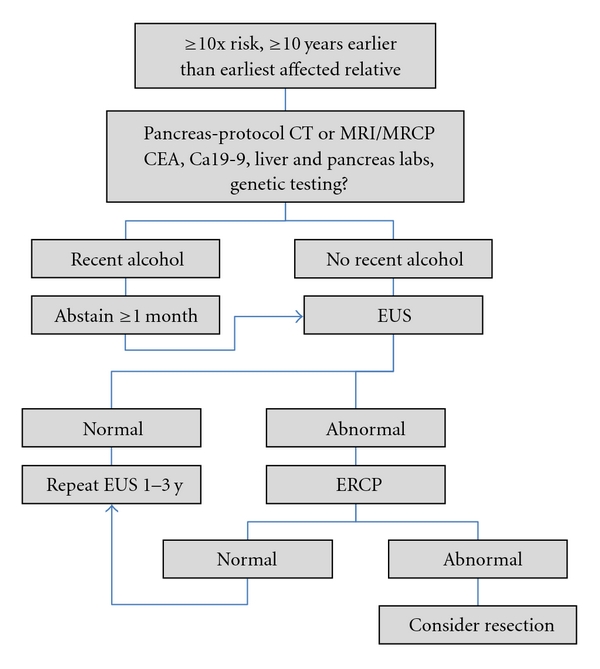
Flow chart for pancreatic cancer screening in high-risk individuals.

Normal EUS findings include homogenous parenchyma and a thin-walled, anechoic main pancreatic duct. Abnormal EUS features that are considered to warrant ERCP followup (Figure 1) include hypoechoic nodules and cysts, echogenic foci, parenchymal heterogeneity, narrowing or dilation of the pancreatic duct, and duct-wall echogenicity [52, 53, 59–61]. Unfortunately, many of these changes are also present in chronic pancreatitis and in recent heavy alcohol intake, as such patients must be stratified accordingly. Concerning ERCP features include saccular deformities or other irregularities of the pancreatic ducts [60, 61].

When to start screening is similarly not universally defined [50]. Applying the screening principles of colorectal cancer by beginning screening for pancreatic cancer 10 years earlier than the youngest affected member in the family is a reasonable starting point. Taking into account, however, the long time between initiation of a PDAC tumor cell and the presence of a PDAC tumor beginning to have metastatic capability (11.7 ± 3.1 years [62]), and taking into account evidence that consecutive generations with FPC die of PC a median of 10 years sooner each subsequent generation [63], and finally taking into account that smokers with FPC develop cancer a decade before nonsmokers, it is reasonable to use judgment in screening selected individuals much earlier. A reasonable screening algorithm is presented in Figure 1.

The treatment of patients who are deemed to have highrisk for cancer or a precancer lesion and who are found to have an abnormality by screening is pancreatectomy. Some recommend routine total pancretectomy, citing multifocality of the disease [59], whereas others recommended partial pancreatectomy [53]. Inadequate data exist to determine which option is associated with the most favorable risk/benefit ratio, but each patient's ability to manage the severe diabetes following total pancreatectomy must be weighed carefully on a case-by-case basis with the risk of leaving behind at-risk pancreas.

## 3. Hepatobiliary Cancers

### 3.1. Hepatocellular Carcinoma

Compared with PC, much less is known about hereditary hepatobiliary cancers. Although the great majority of hepatocellular carcinoma (HCC) cases are sporadic, some data exist to suggest an inherited component of risk. In a study of nearly 5000 HBV carriers from the Liver Unit of Chang-Gung Memorial Hospital and the Government Employee Central Clinics in Taipei, those who had a family history of HCC had a 2.4-fold risk of HCC compared with HBV carriers without a family history of HCC and this risk increased to 5.6-fold if two or more relatives were affected [64]. Similarly, analysis of the Swedish Cancer Registry [65], covering >10 million individuals, revealed a 4.7-fold increased risk of HCC in offspring of patients with HCC. In addition to general familial risk, several specific, known inherited liver diseases have been associated with increased risk for the development of HCC (Table 5).

#### 3.1.1. Specific Diseases with Inherited Predisposition for HCC

Hereditary hemochromatosis (HH) is an autosomal recessive disease associated with various mutations in the *HFE* gene resulting in progressive iron overload in the liver and elsewhere and is associated with an increased risk of HCC. To study whether HH, per se, and not chronic liver disease, is responsible for the increased cancer risk, Fracanzani et al. [66] analyzed the rate HCC in 230 patients with HH and 230 others with noniron-related chronic liver disease, finding a 1.9-fold increased risk of HCC in HH patients after controlling for alcohol abuse, smoking, and family history of cancer. In a modeling study using published life tables, age-specific cancer rates, and DNA studies of archived liver biopsy specimens, Haddow et al. [67] calculated the lifetime risk of HCC in a cohort of 5000 men with the common homozygous C282Y mutation in the *HFE* gene to be 23-fold compared with 1,000,000 normal men. Elmberg et al. [68] studied 1847 Swedish patients with HH and 5973 of their first-degree relatives. Patients with HH had a 20-fold increased risk of HCC, but their first-degree relatives had no increased risk of overall cancers and an only 1.5-fold increased risk of hepatobiliary cancers such as HCC [68]. A recent meta-analysis of 9 studies including 1102 HCC cases and 3766 controls in Europe revealed that the C282Y mutation but not the H63D mutation was associated with HCC in patients with alcoholic cirrhosis [84]. 

 Other hereditary liver diseases have been associated with increased risk of HCC development, such as autoimmune hepatitis (RR 23) [69], porphyria (RR 5–36) [70, 71], *α*1-antitrypsin deficiency (RR 5) [72], progressive familial intrahepatic cholestasis (RR 3.7) [73, 74], glycogen storage disease type 1 (von Gierke disease) (RR unk.) [75], hereditary tyrosinemia type I (RR unk.) [76–78], Wilson's disease (RR unk.) [79], Niemann-Pick disease (RR unk.) [80], Gaucher disease (RR unk.) [81], and hereditary telangieatasias (RR unk.) [82, 83], but these associations are poorly studied due to the rarity of the disease processes.

### 3.2. Bile-Duct and Gallbladder Cancer

In a large, cooperative, case-control series from Milan, Fernandez et al. [12] prospectively followed 740 patients with pancreatic and hepatobiliary cancers compared with 1408 matched control patients and found a family history of gallbladder cancer in 1 of 58 patients with gallbladder cancer and in 2 of 1408 controls, yielding a relative risk of 13.9 but with a wide confidence interval (95% CI 1.2–163.9). Interestingly, a family history of stomach cancer was associated with a nearly 2-fold relative risk of gallbladder cancer [12].

Analysis of the >10-million-person-large Swedish Cancer Registry revealed a 5.2-fold increased risk of gallbladder cancer in the offspring of patients with gallbladder cancer [65]. There was a similar (3.8-fold), but only borderline significant, risk of extrahepatic bile-duct cancer when a family history of maternal ovarian cancer was present [65]. 

Several studies have reported an association between a family history of gallstones and gallbladder cancer. In a recent study from China's Shanghai Cancer Institute, Hsing et al. [85] confirmed earlier reports [86] that simply a family history of gallstones conferred an increased risk of gallbladder cancer (2.1-fold [85] to 3.6-fold [86]), even after adjustment for age, gender, marital status, education, smoking, alcohol drinking body mass index, and importantly, the presence of gallstones, which themselves further increase the risk. 

#### 3.2.1. Specific Diseases with Inherited Predisposition for Bile-Duct Cancer

In a cohort of 472 patients from 15 different families with HNPCC, cancer of the biliopancreatic tract was seen in 18 patients, 11 (79%) of which were confirmed as arising in the biliary tree or ampulla of Vater [87]. Despite a >9-fold increased risk of bile-duct cancer in patients with HNPCC [88], routine screening for bile-duct cancer has not been recommended [89], owing in large part to the difficulty in detecting these cancers and their rarity.

Muir-Torre syndrome (MTS) is an autosomal dominant syndrome described in the 1960s [90, 91] that predisposes to sebaceous skin lesions or keratoacanthomas and visceral tumors. MTS is a variant of HNPCC with the majority of germ-line mutations occurring in the MSH2 gene [92]. Several cases of bile-duct and ampullary cancers have been reported in association with MTS [93–95], including a report of a novel missense mutation in the *MSH2* gene [93]. Although screening for biliary cancers is not currently practical, it has been suggested that screening for ampullary cancers in MTS patients would have a favorable risk/benefit ratio [95].

Bile salt export pump deficiency (BSEP), caused by mutations in *ABCB11 *[96], has been associated with bile-duct cancer [97]. In a study of 82 different *ABCB11* mutations in 109 families [74], 19 of 128 patients (15%) with BSEP mutations developed hepatobiliary mutations, but only 2 of the 19 were bile-duct cancers, the remaining being HCC.

## 4. Summary

Among all hereditary cancers of the pancreas, liver, and biliary tree, only those of the pancreas have been studied well enough to allow for recommendations regarding screening and surveillance. While several known but rare forms of hereditary hepatobiliary cancer exist, screening recommendation cannot be made at this time due to the scarcity of available data. In contrast, any individual with a risk of PC estimated to be ≥10-fold should be screened with EUS by an experienced endoscopist in an experienced center after genetic counseling and informed consent, provided that the individual is willing to undergo pancreatectomy. Screening should begin at least 10 years prior to the age of the youngest affected relative and perhaps even earlier for select patients, such as smokers.

## Figures and Tables

**Table 1:** Epidemiological studies of HPC.

Study, year [ref]	Type	Location	No. of patients	Findings
Falk, 1988 [9]	Case control	Louisiana, USA	363	OR 5.25 if FH

Lynch, 1990 [10]	Descriptive	Nebraska, USA	47	10-fold increased occurrence of PC if FH of PC (7.8% versus 0.6%)

Ghadirian, 1991 [11]	Case control	Montréal, Canada	179	13-fold increased occurrence of PC if FH of PC (6.7% versus 0.7%)

Fernandez, 1994 [12]	Case control	Italy	362	4-fold increased occurrence of PC if FH of PC (3.9% versus 1.1%); RR 3 if FH

Dergham, 1997 [13]	Case series	Detroit, USA	81	9% occurrence of PC if FH of PC.

Coughlin, 2000 [14]	Cohort	USA	3751	RR 1.5 (men)–1.7 (women) if FH

Hemminki, 2003 [15]	Cohort	Sweden	21,000	SIR 2.4 if FH (parent)

Klein, 2004 [16]	Registry	Baltimore, USA	5179	SIR 32 if 3 FDR with PCSIR 6.4 if 2 FDR with PCSIR 4.5 if 1 FDR with PC

Permuth-Wey, 2008 [17]	Meta-analysis	USA, Italy, Canada, Japan	6568	RR 3.4 if FH of PC

OR: odds ratio; RR: relative risk; SIR: standardized incidence ratio; FH: family history; FDR: first-degree relative.

**Table 2:** Known inherited syndromes associated with increased risk of HPC.

Syndrome	Genes	Gene function	Rate of PC in syndrome	O/E → risk of PC	Cumulative risk of PC	References
HP	*PRSS1*; *SPINK1 *	Trypsinogen; Protease Inhibitor	8/246 = 3.2% 10/200 = 5.0% 26/418 = 6.2%	8/0.15 → 53 10/0.115 → 87 26/NR → 67	25–70%	[18–20]
FAMMM	*CDKN2/P16*	Tumor suppressor	9/200 = 4.5% 66/466 = 14% 15/656 = 2.3%	6/0.16 → 38^a^ 2/0.03 → 65^b^ 8/0.6 → 13	13–17%	[21–23]
HBOC	*BRCA1; * *BRCA2; * (*PALB2*)	Tumor suppressor	14/1181 = 1.2%	14/4.4 → 5.9 7/1.3 → 8.9^c^	1.2–6.9%	[24–26]
PJ	*STK11/LKB1*	Tumor suppressor	6/240 = 2.5% 4/31 = 13%	NR/NR → 132	5–36%	[27–29]
HNPCC	*MLH1;* *MSH2;* *MSH6; * *PMS2*	DNA mismatch repair	47 cases in 31 families	O/E→ 8.6	3.7%	[30]
CF	*CFTR*	Transmembrane conductance regulator	1/28, 842 = 0.0035%^d^	1/0.4 → 2.6 7/1.7 → 5.3	“Negligible”	[31, 32]
FPC	Unk.	Unk.	2/1253 = 0.16% 4/634 = 0.63% 5/106 = 4.7%	2/0.44 → 4.5 4/0.62 → 6.4 5/0.16 → 32	NR	[33]

NR: not reported; HP: hereditary pancreatitis; FAMMM: familial atypical multiple mole melanoma; HBOC: hereditary breast and ovarian cancer; PJ: Peutz-Jeghers; HNPCC: hereditary nonpolyposis colorectal cancer; FPC: familial pancreas cancer; CF: cystic fibrosis.

^a^Females ≥56 years old.

^b^Females <55 years old.

^c^If outside the ovarian cancer cluster region.

^d^A total of nine patients have subsequently been identified by Maisonneuve et al. [31, 32].

Families, not individuals.

**Table 3:** Comparison of chronic alcoholic and chronic hereditary pancreatitis.

Pancreatitis type	Chronic alcoholic pancreatitis	Chronic hereditary pancreatitis	*P*
Male to female ratio	12.5 : 1	1 : 1	
Age of onset (years)	40	10.5	<.05
Delay in diagnosis (years)	3	14.3	<.05
Presence of pseudocysts	10%	33%	<.05
Presence of pancreatic calcifications	57%	58%	NS
Endocrine insufficiency	70%	50%	NS
Exocrine insufficiency	42%	38%	NS
Need for surgery	41%	50%	NS

Modified from [34].

**Table 4:** Candidates for pancreatic cancer surveillance.

Candidates for PC surveillance (with >10-fold increased risk of PC)
Anyone with ≥3 first-degree relatives with PC
Individuals with ≥3 first-, second-, or third-degree relatives with PC, at least one of whom is a first-degree relative
Anyone with FAMMM, PJS, or HP
Any member of a PJS family
Carriers of mutations of *BRCA1, BRCA2, *or an MMR gene and with at least one first- or second-degree relative with PC
A person with 2 relatives in the same lineage (directly connected) with PC, at least one of whom is a first-degree relative of the candidate
Some people with two first-degree relatives with PC and favorable expert opinion

Modified from [50].

**Table 5:** Inherited diseases of the liver associated with HCC.

Inherited diseases of the liver associated with HCC		
Disease	RR	References
Hereditary hemochromatosis	2–20	[66–68]
Autoimmune hepatitis	23	[69]
Porphyria	5–36	[70, 71]
*α*1-antitrypsin deficiency	5	[72]
Progressive familial intrahepatic cholestasis	Unk.	[73, 74]
Glycogen storage disease type 1 (von Gierke disease)	Unk.	[75]
Hereditary tyrosinemia type I	Unk.	[76–78]
Wilson's disease	Unk.	[79]
Niemann-Pick disease	Unk.	[80]
Gaucher disease	Unk	[81]
Hereditary telangiectasias	Unk.	[82, 83]
